# Morphometric Analysis of Glenopolar Angle of the Scapula in Indian Population

**DOI:** 10.7759/cureus.65189

**Published:** 2024-07-23

**Authors:** Neelam Kumari, Abhijeet Subhash, Padamjeet Panchal

**Affiliations:** 1 Anatomy, All India Institute of Medical Sciences, Patna, Patna, IND; 2 Orthopaedics, Indira Gandhi Institute of Medical Sciences, Patna, Patna, IND

**Keywords:** neer's 1 view, glenoid cavity, anteroposterior view, shoulder fracture dislocation, gleno-humeral articulation, scapula, measurement, glenopolar angle, morphometric, analysis

## Abstract

Background: The glenopolar angle (GPA), usually ranges from 30° to 45°. GPA measures the tilt of the plane of the glenoid cavity in relation to the axis of the body of the scapula passing from the superior pole of the glenoid cavity to the inferior angle of the scapula. It is essential to assess the results of surgeries for dislocated shoulders. Worse long-term outcomes can arise from glenoid misalignment in scapular neck fractures. When evaluating prognosis and planning therapy for shoulder injuries including scapular fractures, GPA assessment is essential. Still, there is a dearth of data on the normal range of GPA and its contributing elements, which calls for more study. The purpose of this study is to determine typical GPA values by utilizing radiographs and a sizable sample of scapular bone specimens.

Methods: In this study, the GPA was assessed in 50 chest radiographs of anteroposterior (AP) view and Neer’s view of individuals as well as 100 dried scapulae of any gender. The mean GPA obtained using the various methods was then statistically compared.

Findings: All scapulae had an average GPA of 42.6°. Twenty-nine scapulae had GPA observations higher than 45°, with an average of 47.2°. Twenty-seven scapulae had GPA measurements below 40°, averaging 37.3°. Right-sided 62 scapulae with an average GPA of 43.1° were present. Thirty-eight of left-side scapulae had a GPA of 41.7° on average. It was determined that the 1.6° mean difference in GPA between the two groups was not statistically significant. The Kolmogorov-Smirnov test verified that the GPA data had a normal distribution. The homogeneity of variances across various measuring techniques was confirmed using Levene’s test. The average GPA measured using the dry scapula approach was 42.6°, the average GPA measured using the AP view was 39.8°, and the average GPA measured using the Neer I view was 42.3°. The GPA means from these three approaches differed considerably (p=0.0014) according to a one-way Analysis of Variance (ANOVA). Fisher’s least significant difference post hoc testing showed that the scapular bone specimens and the Neer I view revealed significantly higher GPA values than AP shoulder radiographs. The GPA values obtained from the osteological group and the Neer I perspective had a mean difference of 0.21°, which was practically identical and suggested that there was no statistically significant difference between these approaches.

In summary: In order to diagnose and treat a variety of shoulder joint diseases, this study estimates the normal values of scapular GPA. Furthermore, it offers support for improved implant design in the context of Indian shoulder joint replacement and repair. Using every measurement technique, the GPA values on the right and left sides did not differ significantly. GPA results from various measuring methods varied significantly, which emphasizes the significance of methodological consistency in clinical and research settings.

## Introduction

The glenopolar angle (GPA) is the angle formed at the superior pole of the glenoid cavity. It is formed by the intersection of two lines between the line (AB) and the line (AC) connecting the most superior pole of the glenoid cavity with the most inferior point (C) of the scapular body at an inferior angle. Line AB represents the glenoid plane, which extends from the superior pole (A) to the inferior pole (B) of the glenoid cavity in the scapula. Line AC represents the scapular body axis, which extends from the superior pole (A) of the glenoid cavity to the inferior angle (C) of the scapula. The GPA, which usually varies from 30° to 45°, is an assessment of the glenoid’s tilt about an anteroposterior axis perpendicular to the scapular body plane. It measures how obliquity there is between the scapular body and the glenoid articular surface [1]. Bestard et al. considered the normal range of GPA between 30°-45° and a useful indicator of scapular neck fracture displacement [2, 3]. Romero et al. [4] reported that scapular neck fractures with malalignment have a poorer long-term outcome than cases without glenoid malalignment did, as measured using the GPA. In addition, Labler et al. recommend operative treatment with a displacement of a scapular neck fracture exceeding 25 mm or reducing the GPA below 3° as an indirect sign of rupture of the associated ligaments [5]. Glenopolar and glenoid inclination angles are important when deciding on the treatment and prognosis of a floating shoulder [5]. Kim et al. emphasize GPA evaluation while planning the treatment of floating shoulder and follow-up [3]. Cole et al. proposed that the GPA is a critical factor influencing the efficacy of surgical treatments for body and neck fractures of the scapula [6]. However, few authors as per the searched literature, dealt with the normal range of GPA values and the effect of various techniques applied for GPA measurement on the existing GPA values. So main aim of this article was to find out the normal range values of GPA on a large number of bone specimens of scapula and radiographs by different methods and the impact of different measurement methods on the GPA.

## Materials and methods

**Figure 1:**
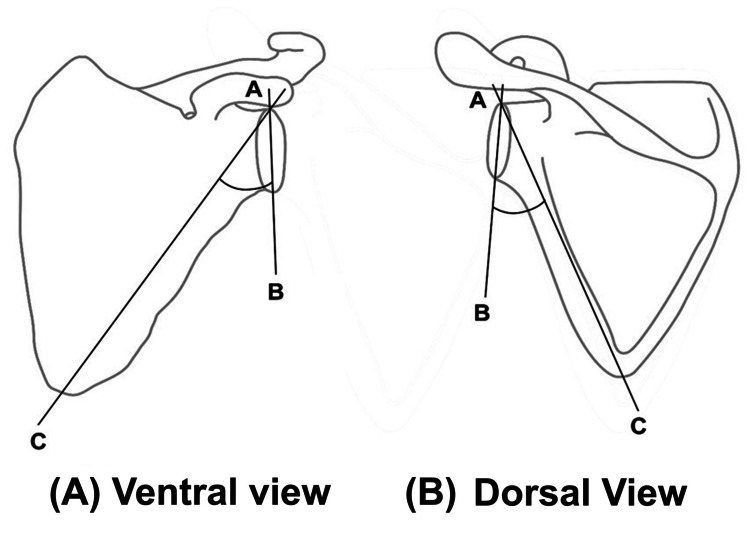
The GPA of the left scapula: (A) ventral view and (B) dorsal view. Line (AB) connects the most superior point (A) on the upper margin and the most inferior point (B) on the lower margin of the glenoid cavity. Line (AC) connecting the most superior margin of the glenoid cavity with the most inferior point (C) present on the inferior angle of the body of the scapula.

The investigation was carried out after the request for authorization to conduct the study was granted by the institutional ethics committee (approved number 604/IEC/IGIMS dated 18/12/2018). There were two main steps that were carried out throughout the research. Following the first phase, which consisted of capturing 50 individual radiograms of the shoulder area of both genders, the second step entailed utilizing 100 cadaveric-dried human scapulae in order to examine the GPA. This was done in order to determine the GPA by a method as suggested by Bestard et al. (Figure 1) [2].

Patients were chosen at random from among those who attended the radiology department. They were recommended to have plain radiography done because of their history of shoulder pain and present symptoms, which included restricted mobility and stiffness in the shoulder joint. A standard approach was followed to acquire conventional radiographs of the shoulder area. To enable the Neer’s view radiograph, participants were advised to stand straight or lie on their backs. They were requested to lift their arm and turn their hand upward, with the thumb facing downward. The Neer’s view is a particular radiographic projection that concentrates on the proximal humerus and the glenohumeral joint. Neer’s perspective takes into account radiographs when there is an overlap between the glenoid anterior and posterior lips. Additionally, a radiograph was acquired from an anteroposterior orientation. X-ray beam should be aligned at a 90° angle to the plane of the scapula, aiming directly toward the center of the scapula. To achieve this perpendicular alignment, the X-ray beam is angled at approximately 35° from the sagittal plane. This specific angle helps to minimize superimposition of other structures and soft tissues, thereby providing a clearer view of the scapular body. The resulting radiograph demonstrates the scapula in true AP projection, where the body of the scapula appears broad and flat.

**Figure 2:**
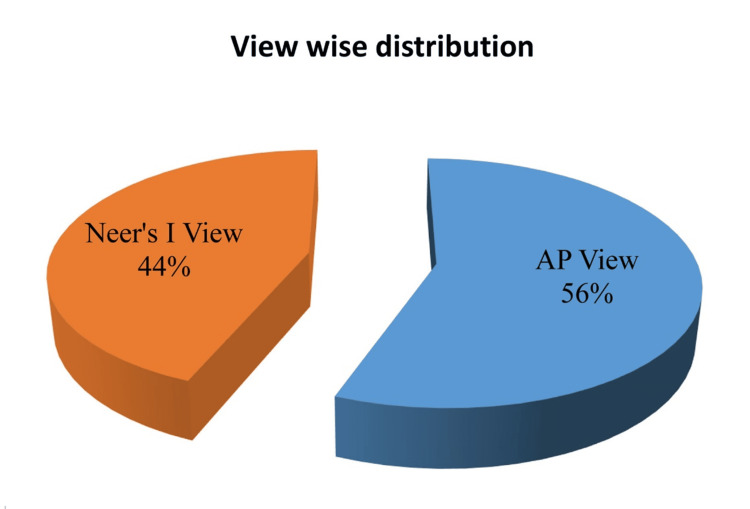
View-wise percent distribution of radiographs. AP view: anteroposterior view, Neer’s I view: Neer method is an X-ray examination of the shoulder using tangential projection of central ray at 10 to 15° angulation.

The GPA measurement on digital radiographs was done using Angulus Software Version 3.1.5. A total of 50 radiographs were used for GPA assessment. There were a total of 28 radiographs taken in the anteroposterior view and 22 radiographs taken in Neer’s I view (Figure 2).

A total of 100 fully developed non-paired human scapulae, of undetermined age and gender, were obtained from the bone repository of the anatomy department at the institution. Bones that have not ossified or have incomplete bone formation; bones that show signs of surgical procedures, or alternation such as implants, grafts, or other orthopedic interventions; radiographs that are of poor quality or unclear; and any scapula bone that has been significantly destroyed structurally, destroyed as a result of trauma, degenerative disease, or surgical intervention were excluded from this research. In compliance with Neer’s view standards, radiographs without the superimposition of glenoid lips were excluded to prevent projection errors and ensure precise capturing of the glenohumeral joint line. The Neer I view radiographs meet specific standards making the data more accurate and comparable.

Statistics

The Prism 5 application (GraphPad Software) was used for statistical analysis. The normal distribution of data was verified by using the Kolmogorov-Smirnov test. The mean GPA, range of GPA values, and standard deviation were utilized to describe the GPA metrics. A one-way analysis of variance was used to compare the radiographic measurements to the bone specimens. The Fisher’s least significant difference (LSD) test was used for a post hoc analysis. One-sample (pair) t-tests to compare the quantitative data on both sides.

## Results

**Figure 3:**
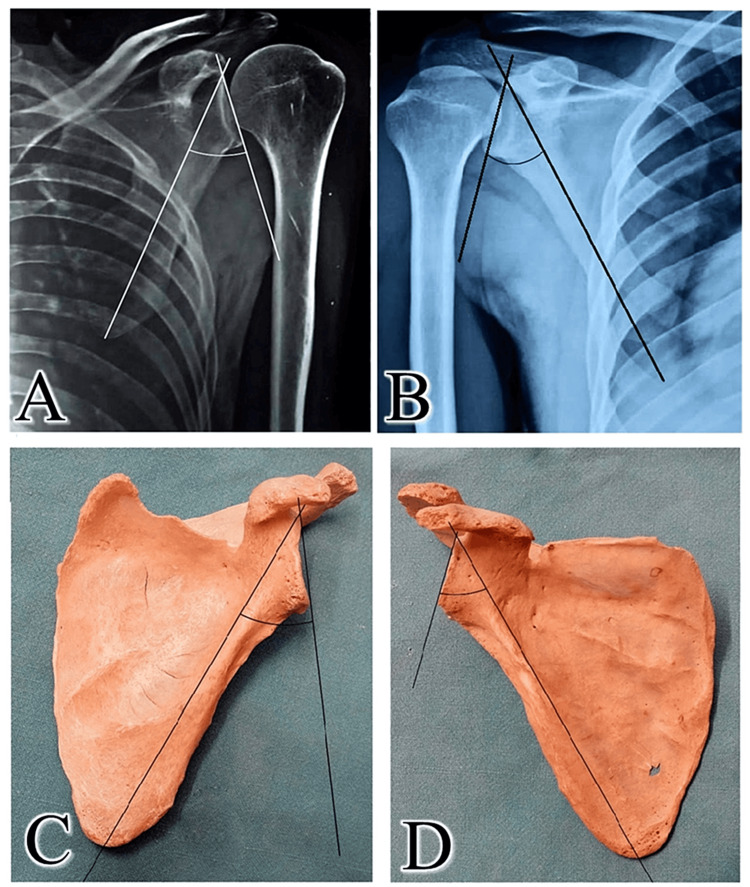
The GPA angle of the scapula acquired by various methods. A) left scapula (Neer I view) in radiograph of the shoulder region; (B) right scapula (AP view) in the radiograph of the shoulder region; (C) ventral aspect of left scapula; and (D) ventral aspect of right scapula. GPA: glenopolar angle; AP: anteroposterior; Neer’s I view: Neer method is an X-ray examination of the shoulder using tangential projection of central ray at 10 to 15° angulation.

The Kolmogorov-Smirnov test confirmed that the GPA data obtained from different methods followed a normal distribution. Additionally, the homogeneity of variances was verified using Levene’s test. The GPA estimated in radiographs was compared with dry scapula GPA measurements (Figure 3).

Anatomical measurements was done on the 100 human scapulae revealed a GPA value ranging from 30° to 56° with an average of 42.6°. GPA measured more than 45° in 29 scapulae, having a mean GPA of 47.2°, ranging between 46°- 49° and GPA estimated at less than 40 in 27 scapulae, having a mean GPA of 37.3°, ranging from 30° to 39°. Among 100 scapulae, 62 scapulae were on the right side, having a mean GPA of 43.1°, which ranges from 30° to 49°, and 38 scapulae were on the left side, having a mean GPA of 41.7°, ranging between 35° and 48° (Table 1).

**Table 1:** Variables of GPA measurements on dry bone. GPA: glenopolar angle

Parameters	Number of Scapula	Mean GPA	Range	
Total	100	42.6°	30°- 49°	
GPA>45°	29	47.2°	46°- 49°	
GPA<40°	27	37.3°	30°- 39°	
Right	62	43.1°	30°- 49°	
				Left	38	41.7°	35°- 48°		

The mean difference in values between the GPA identified on the right side and the GPA on the left side was on average 1.6°.

Radiological study measurements were made on 50 radiographs, and the average GPA measured was 39.8°±5.14 (range 29-48°). The average GPA calculated on 28 AP view was 39.8°, ranging from 29° to 46°. The average GPA counted on 22 Neer I views was 42.3° (36-48°). A comparison of individual methods and side differences in one-way ANOVA rejects the null hypothesis about the simultaneous equality of three different methods of GPA measurements (p=0.0014) (Table 2).

**Table 2:** ANOVA summary table. p-value ≤ 0.05 is considered statistically significant. F-statistic: ratio of the variance between the groups to the variance within the groups, p: probability

Source of Variation	Sum of Squares	Degree of Freedom	Mean Square	F-statistic	p-value	F-Statistic Critical Value
Between groups	261.7633	2	130.8816	6.79	0.0014	3.05
Within groups	3254.981	169	19.26			
Total	3516.744	171				

The calculated F-statistic (6.79) is greater than the critical value (3.05), and the p-value (0.0014) is less than 0.05. Thus, it may be concluded that there are significant differences between the GPA means of the three group methods. The more detailed LSD post hoc tests indicate that significantly lower GPA values from AP shoulder radiographs were obtained against higher GPA values provided by anatomical bone specimens, Neer I view. The LSD value helps determine if the difference between any two-group means is statistically significant (Table 3).

**Table 3:** LSD post hoc test results LSD: least significant difference test done to distinguish different methods group means

Group Comparison	Mean Difference	LSD Value	Statistically Significant (Yes/No)
Scapula bone vs. AP view	2.75	1.618	Yes
Scapula bone vs. Neer’s I view	0.21	1.618	No
AP view vs. Neer’s I view	2.54	1.618	Yes

When the GPA obtained from the osteological study was compared to Neer’s I view, the mean difference or absolute value was 0.21. This suggests that the GPA values obtained from both these methods are almost equal, with no statistical significance. The right and left side differences were found to be statistically insignificant for the GPA values that were compared by all measurement methods.

## Discussion

The scapula has an essential role in the function of the arm. The congruency of the scapula against the ribs stabilizes the upper extremity against the thorax. The scapula connects the upper extremity to the axial skeleton via the glenohumeral joint, the acromioclavicular joint, and the sternoclavicular joint. The scapula possesses several muscle attachments that play a vital role in both the movement and stabilization of the scapula and shoulder joint. The scapula plays an essential role in the movement of the shoulder girdle. Its peculiar shape has always been a point of attraction to many anatomists [7]. As we know, scapular fractures are rare injuries and represent only 3%-5% of shoulder girdle injuries and less than 1% of all fractures [8-10].

Displacement of more than 25 mm scapular neck fracture or reduction of the GPA of less than 30° was an indirect sign of associated ruptured ligaments. Therefore, Non-surgical therapy is appropriate for minor fracture displacement when ligaments remain intact or when the angle of displacement (GPA) exceeds 30°. Ludwig Labler et al. used GPA to compare the clinical and functional outcomes of operative and non-operative treatment of floating shoulder injury [5]. Consequently, GPA is crucial in determining whether conservative or surgical intervention is warranted [11-15].

Hardegger et al. established a classification system for scapular neck fractures, dividing them into anatomical and surgical neck fractures [8]. The scapula surgical neck fracture traverses the spinoglenoid notch, connecting the supraspinous notch to the inferior pole of the glenoid cavity dorsally. In contrast, the anatomical scapular neck is lateral to the base of the coracoid process. This distinction is important because the anatomical neck is more lateral and involves a different section of the scapula than the surgical neck, usually considered for surgical interventions. Scapular neck fractures can potentially disrupt the relationship between the glenohumeral and acromial structures, resulting in functional imbalance. Anteroposterior radiographs taken in the scapular plane were employed to measure the GPA, while the patient's hand was positioned on the iliac crest for reference. Subsequent researchers have replicated this methodology [16-17]. Romero et al. elucidated that the clinical outcome of scapular neck fractures can vary due to the potential for permanent malalignment of the glenoid neck [4]. They utilized the GPA method to assess malalignment in scapular neck fractures, as Bestard et al. suggested [2]. This angle is defined as an angle between the line connecting the most superior margin and the inferior point on the margin of the glenoid cavity and the line connecting the most superior edge of the glenoid cavity to the inferior most point of the scapular body [2,3]. They noted that fractures of the clavicle or dislocation of the acromioclavicular joint could occur with or without significant displacement of the glenoid. In such instances, a glenopolar joint angle measuring less than 20° could serve as an identifying marker.

Kim et al. determined a GPA of 35°, in 3D CT reconstructions. The researchers introduced the Constant Murley score (CMS), a scale ranging from 0 to 100, which encompasses various parameters to evaluate pain levels and the ability to perform daily activities affected by the GPA of the scapula [3]. They found that patients with a GPA above 30° tended to have better CMS for the affected shoulder compared to those with a GPA below 30°. The higher CMS indicates that affected patients experienced less pain, had better functional ability in daily activities, and demonstrated better shoulder strength and range of motion compared to those with a GPA below 30°. Additionally, the GPA of the affected shoulder and its change relative to the unaffected shoulder were correlated with the floating shoulder’s clinical outcome [15,18,19]. Izadpanah et al. discovered an excellent correlation between the final GPA angle and the CMS. In their study, all cases involved clavicle stabilization with minimal displacement of the scapular neck [20]. Despite the abundance of studies utilizing the GPA to forecast the prognosis of scapular fractures [2, 21-25] and the consensus on normal GPA values as established by Bestard et al. [2], the methodologies used to determine these norms are not explicitly outlined. Subsequent authors measured the GPA using different radiological views, yet none addressed the efficacy of these methods for calculating GPA [2, 21-24]. Additionally, the impact of projection on GPA values was not discussed [26]. Only Wijdicks et al. conducted experimental research explaining that GPA values change with scapular rotation. They observed a significant variation in the measured angle with increasing degrees of scapular rotation [27]. Notably, there was no significant difference in GPA from 0° to 10°, but a significant difference was observed from 0° to varying degrees of rotation starting at 20°, a finding later validated by other studies [28,29].

There were certain restrictions on this research. The precision of the data could be impacted by variations in patient placement and X-ray beam alignment. Measurement errors due to software limitations or user handling could affect the GPA values. The reliability and reproducibility of GPA measures are enhanced by precise and consistent projections. Differences in measured GPA values resulting from variations in the radiography method can compromise the validity of comparisons between the afflicted and non-affected sides. This mostly affects AP chest and Neer I radiographs. The standard of radiographs has implications for measuring GPA. 3D CT reconstructions of afflicted and unaffected sides can overcome these issues. However, its drawbacks include significant exposure to radiation and expense.

## Conclusions

The mean GPA in dry bone was measured at 42.6°, with a range of 30° to 49°. Remarkably, the mean GPA value derived from Neer’s I view radiogram was 42.3°, but it registered as 39.8° in the AP view radiogram. These results indicate that the GPA measurement obtained from dry bone closely corresponded to the findings from Neer’s I view radiogram when compared to the AP view. Therefore, while assessing and strategizing for surgical results in situations of shoulder dislocation, it is advisable to use the Neer I perspective. Moreover, the increased concordance between GPA values obtained from dry bone and Neer’s I view radiogram suggests a more precise depiction of the true GPA in clinical settings. Thus, employing Neer’s I-view radiogram for GPA assessment has the potential to enhance surgical planning and improve results in cases of shoulder dislocation. The precision of GPA measurements can be affected by patient placement and X-ray beam alignment, software limitations, and radiographic variations. 3D CT reconstructions offer accuracy but involve high radiation exposure and cost.
